# Transition of clinical biomarker status from childhood into adolescence–A prospective study in children from eight European countries

**DOI:** 10.1371/journal.pone.0311180

**Published:** 2025-02-03

**Authors:** Anna Floegel, Paola Russo, Toomas Veidebaum, Michael Tornaritis, Dénes Molnár, Lauren Lissner, Stefaan De Henauw, Luis A. Moreno, Wolfgang Ahrens, Manuela Marron, Claudia Börnhorst

**Affiliations:** 1 Leibniz Institute for Prevention Research and Epidemiology—BIPS, Bremen, Germany; 2 Section of Dietetics, Faculty of Agriculture and Food Sciences, Hochschule Neubrandenburg–University of Applied Sciences, Neubrandenburg, Germany; 3 Institute of Food Sciences, National Research Council, Avellino, Italy; 4 National Institute for Health Development, Estonian Centre of Behavioral and Health Sciences, Tallinn, Estonia; 5 Research and Education Institute of Child Health, Strovolos, Cyprus; 6 Department of Pediatrics, Medical School, University of Pécs, Pécs, Hungary; 7 Section for Epidemiology and Social Medicine, Department of Public Health and Community Medicine, Institute of Medicine, Sahlgrenska Academy, University of Gothenburg, Gothenburg, Sweden; 8 Department of Paediatrics, Institute of Clinical Sciences, Sahlgrenska Academy at Gothenburg University, Gothenburg, Sweden; 9 Department of Public Health, Ghent University, Ghent, Belgium; 10 GENUD (Growth, Exercise, Nutrition and Development) Research Group, Faculty of Health Sciences, Universidad de Zaragoza, Instituto Agroalimentario de Aragón (IA2), Instituto de Investigación Sanitaria Aragón (IIS Aragón), Zaragoza, Spain; 11 Centro de Investigación Biomédica en Red de Fisiopatología de la Obesidad y Nutrición (CIBERObn), Madrid, Spain; 12 Institute of Statistics, Faculty of Mathematics and Computer Science, University of Bremen, Bremen, Germany; Xuzhou Central Hospital, The Xuzhou School of Clinical Medicine of Nanjing Medical University, CHINA

## Abstract

**Purpose:**

Understanding factors influencing clinical biomarkers is important for the prevention of chronic disease. This study aimed to estimate transitions of biomarker status from childhood to adolescence and to identify determinants of biomarker status in early life in a prospective children cohort.

**Subjects and methods:**

Our sample comprised 1295 children participating in the baseline (2007/08) and second follow-up examination (2013/14) of the multi-center IDEFICS (**I**dentification and prevention of **D**ietary- and lifestyle-induced health **EF**fects **I**n **C**hildren and infant**S**)/I.Family study. Clinical blood biomarkers including glycated hemoglobin A1c (HbA1c), high-density lipoprotein cholesterol (HDL-cholesterol), triglycerides, C-reactive protein (CRP), interleukin 6, ferritin, leptin and insulin-like growth factor 1 (IGF-1) were repeatedly measured in 2007/2008 (age range: 3.0 to <10.0 years) and in 2013/2014. Latent transition analysis was used to estimate biomarker statuses and transition probabilities; determinants of biomarker status were estimated using mixed-effects models.

**Results:**

Four distinct biomarker statuses were identified: (1) “normal” (all biomarkers low/medium; except HDL-cholesterol; reference), (2) “low leptin/IGF-1/HbA1c”, (3) “dyslipidemia/high leptin” and (4) “inflammation”. Children classified as “low leptin/IGF-1/HbA1c” at baseline were most likely to stay in this status (89.8%) or to change to the “normal” status (10%) during follow-up. Compared to “normal” children, children classified as “low leptin/IGF-1/HbA1c” were less likely to have a family history of diabetes (0.26 [0.08;0.86]; odds ratio (OR) and 95% confidence interval) or hypertension (0.53 [0.29;0.99]) and the children (0.32 [0.27;0.38]) as well as their mothers (0.93 [0.88;0.98]) had a lower BMI. Children from families with low/medium education had a 55% [9%-119%] higher risk of being in the “dyslipidemia/high leptin” and 49% [1%-121%] higher risk of being in the “inflammation” status as compared to children in the “normal” status. Membership in a sports club reduced the latter risks by 28% [2%-47%] and 40% [17%-56%], respectively.

**Conclusions:**

European children showed distinct phenotypes for the investigated biomarkers. Especially parental characteristics like a family history of diabetes or hypertension, a high maternal BMI, or low/medium education were associated with unfavorable biomarker status in children.

## Introduction

The childhood obesity epidemic is spreading worldwide with huge implications for reduced quality of life, premature onset of chronic diseases, and high health economy burden [1]. According to the World Health Organization more than 390 million children and adolescents between 5 and 19 years were overweight and of them 160 million were diagnosed with obesity in 2022 [1]. If the increasing current trends for children and adults continue, health economy costs are expected to increase by four-fold in high income countries, and 12- to 25-fold in low- and middle income countries from 2019 to 2060 [2]. Overweight and obesity in childhood lead to further metabolic disturbances and have been linked to higher risk and early onset of chronic diseases such as type 2 diabetes and cardiovascular diseases [1].

In turn, changes in biomarker levels, that reflect pathophysiological processes, usually precede chronic diseases and may necessitate changes in lifestyle or medical therapies in children [3]. Regular screening of clinical biomarkers in children at risk may help to assess metabolic alterations as early as possible and offers the opportunity to prevent chronic diseases in the long run [4]. Relevant metabolic pathways and changes in biomarker concentrations that are linked to childhood obesity and chronic disease risk include the following: 1.) Dyslipidemia which implies disturbed lipid and lipoprotein metabolism and consequently elevated blood concentrations of triglycerides and reduced concentrations of high density lipoprotein (HDL) cholesterol [5]. 2.) Disrupted glucose metabolism and insulin resistance, which may be diagnosed by elevated fasting glucose concentrations or glycated hemoglobin A1c (HbA1c) [6]. 3.) Chronic low-grade inflammation which results in a rise in inflammatory biomarkers such as interleukin-6 (IL-6) and consequently C-reactive protein (CRP) [7]. 4.) Changes in concentrations of adipokines such as leptin which may be a link between obesity and other metabolic disturbances, e.g. via the proinflammatory properties of leptin it may be involved in development of dyslipidemia, insulin resistance and cardiovascular complications [8]. 5.) Onset of the metabolic syndrome which is a combination of abdominal obesity, hypertension, dyslipidemia and impaired fasting glucose [5]. 6.) And last but not least, considering the development and growth of children other biomarkers that may be relevant for metabolic outcomes during childhood include growth related biomarkers such as Insulin like growth factor 1 (IGF-1) in line with those reflecting nutritional status [9].

As the aforementioned pathophysiological consequences often occur jointly such as in metabolic syndrome, it is important to measure multiple biomarkers and their synergies rather than to rely on single clinical biomarkers [3]. Most previous studies that investigated multiple biomarkers in children were focused on prevalent disease conditions [10], measured biomarkers of exposure [11] and/or used a cross-sectional design [12]. Smaller longitudinal studies reported progression of dyslipidemia during childhood particularly in children with obesity [13–15]. A birth cohort in the United States (US) observed distinct leptin trajectories in children that were linked to higher risk of obesity in adolescence [16]. In the same cohort, determinants of selected metabolic biomarkers included sex, weight and puberty status and ethnicity [17]. Another study reported highest CRP concentrations for obese children that stayed obese from primary school to puberty, and lower CRP concentrations in children that were able to change from overweight to normal weight during childhood [18]. In the same study CRP concentrations were positively correlated with leptin concentrations, and inversely associated with HDL-cholesterol [18]. A recent study in toddlers in Tanzania found that higher IGF-1 and lower ferritin concentrations at age 12 months were associated with greater length, weight and head circumference at age 18 months [19]. To sum up, few studies have been conducted that investigated longitudinal transitions of multiple clinical biomarkers from childhood into adolescence. To our knowledge there is no such study available yet in a large children cohort covering several European countries.

Therefore, we aimed to study changes and determinants of multiple clinical biomarkers over a six year time span from early childhood into adolescence in the large European IDEFICS/I.Family children cohort covering the age span from 2 to 15 years. We identified distinct biomarker statuses and their transition probabilities from childhood to adolescence as well as risk factors for adverse biomarker status. The results of the present study may help to better understand metabolic changes during childhood and add evidence for scientists and practitioners to develop individual and target group specific prevention strategies.

## Material and methods

### Study population

IDEFICS (Identification and Prevention of Dietary- and Lifestyle-Induced Health Effects in Children and Infants)/I.Family is a cohort study that investigates the causes of diet- and lifestyle-related diseases in children, adolescents and their families. It is a multi-center population-based study that was conducted in eight European countries (Belgium, Cyprus, Estonia, Germany, Hungary, Italy, Spain and Sweden), with a total of 16,229 children aged 2 to 9 years participating in the baseline survey in 2007/2008. In each country two or more communities with similar socio-demographic profiles were selected where all children attending kindergartens and primary schools were eligible. Via these settings parents were approached. The survey involved interviews with parents about their children’s lifestyle habits and dietary intakes, physical examinations of the children, and the collection of blood samples using standardized protocols, assessment methods and procedures across countries. A first follow-up examination was conducted in 2009/2010 with 13,596 children participating. A second follow-up examination took place in 2013/2014, with 7,105 children participating who were already part of the study at baseline or first follow-up. Fasting blood samples were collected at baseline and during both follow-up waves (see S1 File).

Parents provided written informed consent before children entered the study. In addition, children aged 12 and older gave written consent for the examinations and sample collections, while younger children gave oral consent. The studies involving humans were approved by the appropriate institutional review boards of all eight study centers (1. Belgium: Ethics Committee of the Gent University Hospital, 15 October 2007, ref: No. EC UZG 2007/243 and 19 February 2013, No. B670201316342; 2. Cyprus: Cyprus National Bioethics Committee, 12 July 2007, ref: No. EEBK/EM/2007/16 and 21 February 2013, No. EEBK/ETI/2012/33; 3. Estonia: Tallinn Medical Research Ethics Committee (TMREC), 14 June 2007, ref: No. 1093 and 17 January 2013, No. 128; 4. Germany: Ethic Commission of the University of Bremen, 16 January 2007 and 11 December 2012; 5. Hungary: Medical Research Council, 21 June 2007, ref: 22-156/2007-1018EKU and 18 December 2012, 4536/2013/EKU; 6. Italy: Ethics Committee of the Local Health Authority (ASL) in Avellino, 19 June 2007, ref: No. 2/CE and 18 September 2012, No. 12/12; 7. Spain: Ethics Committee for Clinical Research of Aragon (CEICA), 20 June 2007, ref: No. PI07/13 and 13 February 2013, No. PI13/0012; 8. Sweden: Regional Ethics Research Board in Gothenburg, 30 July 2007, ref: No. 264–07 and 10 January 2013, No. 927–12). The studies were conducted in accordance with the local legislation and institutional requirements. Further details on the study design and objectives can be found in Ahrens et al [20].

### Selection of biomarkers

Our latent transition analysis allowed the inclusion of up to eight biomarkers. Models with a higher number did not converge. These eight biomarkers were selected to cover different pathways of metabolism and based on their availability in the cohort. We included the following biomarkers in our analyses in order to cover different pathways and based on availability in the cohort: HbA1c (glucose metabolism), HDL-cholesterol and triglycerides (lipid metabolism), CRP and IL-6 (inflammation), ferritin (acute phase protein & biomarker of body iron storage), leptin (adipokine), and IGF-1 (growth & metabolic effects).

### Blood sample collection and biomarker measurements

Fasting blood samples were collected at baseline and during two follow-up (FU) waves. In case parents of young children refused venipuncture, capillary blood was collected instead. At baseline serum HDL-cholesterol and triglycerides were assessed using a point-of-care analyzer (Cholestech LDX, Cholestech Corp., Hayward, CA, USA). In the second FU wave, an enzymatic colorimetric test was applied to measure serum HDL-cholesterol and triglycerides (Cobas c701, Roche Diagnostics GmbH, Mannheim, Germany). We conducted validation measurements to confirm that the different analytical methods produced similar and robust results. The other biomarkers were measured with the same methods at all waves. HbA1c was measured in EDTA whole blood with high performance liquid chromatography (Variant, Biorad, Munich, Germany). Serum adipokines such as leptin and inflammatory biomarkers including CRP and IL-6 were measured with electrochemiluminescence technology (Protein Multiplex, Meso Scale Discovery). Ferritin was quantified with immuno-nephelometry (Siemens Healthcare Diagnostics Products GmbH, Marburg, Germany). IGF-1 was measured with a chemiluminescence assay (Immunodiagnostic Systems GmbH, Frankfurt, Germany). All blood samples were analyzed centrally in accredited laboratories.

### Determinants of biomarker statuses

According to previous literature, several potential determinants of biomarker statuses were chosen a priori that are described in detail in the S1 File.

We considered the following lifestyle factors: BMI z-score of the child was derived according to the extended criteria of the International Obesity Task Force (IOTF) [21], consumption frequencies of fruits and vegetables (times/day), consumption frequencies of processed foods (times/day), a psychosocial well-being score (range: 0–48; a higher score indicating a higher well-being), membership in a sports club (yes vs no) and the number of media devices in the child’s bedroom (modeled as 0 vs ≥1).

In addition, the following non-modifiable risk factors (from a child’s perspective) were considered: Age of the child, sex, country of residence, highest educational level of parents according to the International Standard Classification of Education (ISCED; modeled as low/medium vs high) [22], maternal BMI, family history of diseases (hypertension, dyslipidemia and type 2 diabetes; yes vs no), birth weight (g), total breast-feeding duration (in months) and pubertal status (yes vs no).

### Eligibility criteria/ analysis dataset

We used data from baseline 2007/08 and the second FU wave 2013/14 as most biomarkers were measured at these two time points (in the following referred to as T0 and FU). Laboratory measurements from non-fasting blood samples were not considered (370 measurements at T0, 710 at FU). We included children with a maximum of three missing biomarker values at each wave. Children taking medications that might have influenced our parameters of interest were excluded, i.e. children being treated for type1/type2 diabetes (Anatomical Therapeutic Chemical (ATC) codes: A10A, A10B, A10X), elevated blood lipids (C10), obesity (A08), rheumatism/inflammation (non-steroidal; M01), inflammation/asthma (steroidal; H02), anemia (B03) or with growth hormones (H01) were identified based on ATC codes and excluded (N = 184 in T0, N = 86 in FU).

**Fig 1:**
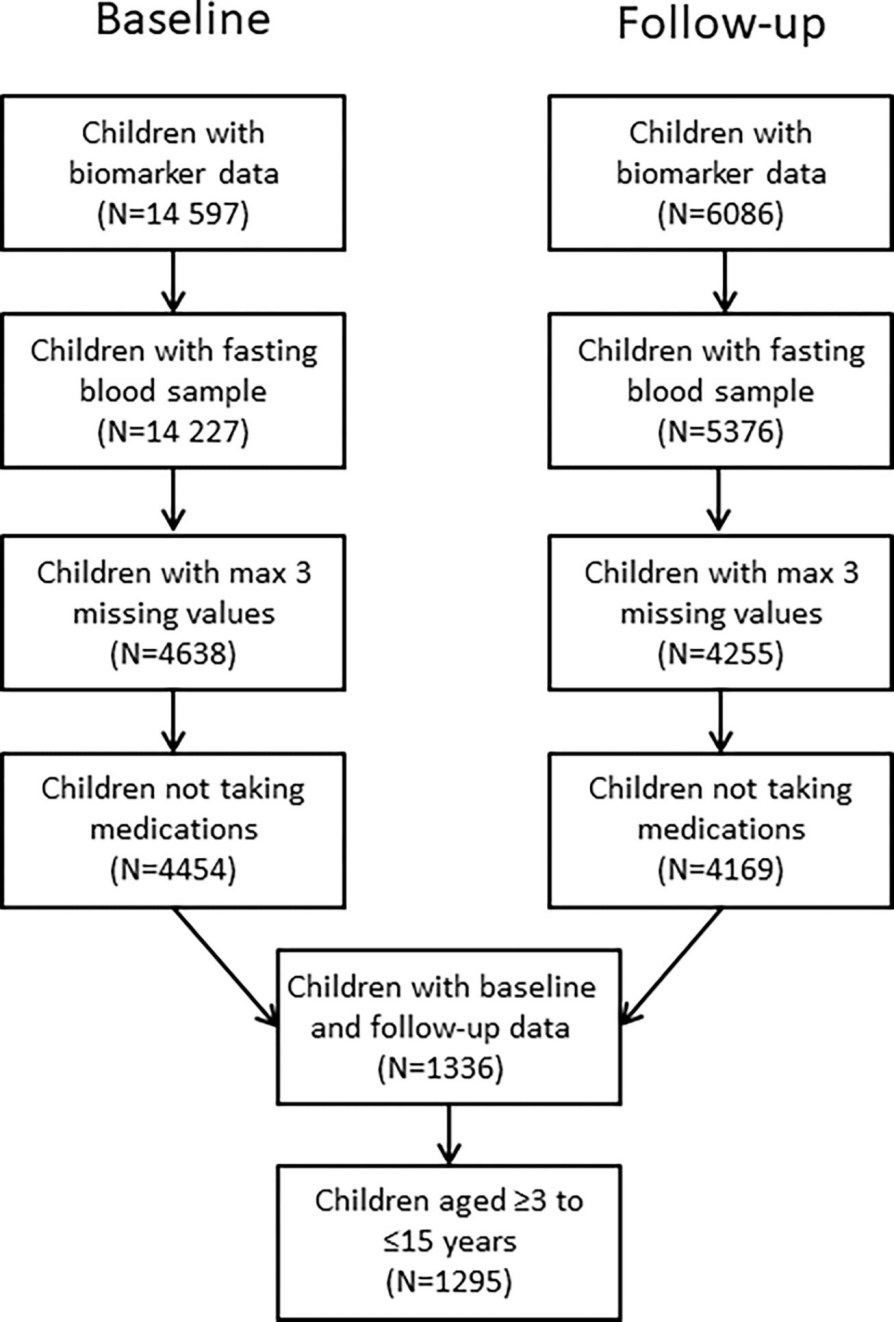
Flow chart of selection of the final study sample in the IDEFICS/I.Family cohort.

Our final analysis dataset included 1295 children aged ≥3 to ≤15 years across the two time points. The flow chart showing the selection process leading to the final analysis sample is presented in Fig 1.

### Statistical analysis

For the eight selected biomarkers, the 15th, and 85th age group- and sex-specific percentiles were calculated as described in the S1 File. Percentiles were calculated separately for the two examination waves as biomarker distributions differed which may be explained by the partly use of different laboratory assessment methods at the two waves. Based on the categorized biomarkers, latent transition analysis (LTA) [23] was conducted to identify groups of children with distinct biomarker statuses (latent groups) and to estimate (a) probabilities (prevalence) for latent biomarker statuses at T0 and FU, (b) probabilities for transitions between latent statuses from T0 to FU as well as (c) item-response probabilities conditional on latent status membership (i.e. probabilities of showing “low”, “medium” or “high” biomarker levels in the different latent statuses). LTA models can handle missing data assuming data to be missing at random such that missing data were not imputed in this step. The statistical analyses are further detailed in the S1 File.

We estimated models with 2 up to 7 latent statuses. Based on the Akaike Information Criterion (AIC) and Bayesian Information Criterion (BIC) different models turned out to have the best fit (the 7-status model for the AIC and 3-status/ 4-status models with almost identical BIC, respectively; see S1 File). Model selection was also guided by theoretical considerations, i.e. which statuses are clearly distinguishable from each other as indicated by different patterns of item-response probabilities and interpretable. We finally selected the 4-status model due to good fit with regard to both, AIC and BIC, interpretability and group sizes.

The four latent biomarker statuses were labeled and characterized as follows (see Table 1):

**Normal:** high probability of all clinical biomarkers being within the low or medium category, except medium to high HDL-cholesterol levels

**Inflammation:** higher than normal probability of having high CRP, IL-6, ferritin and leptin

**Dyslipidemia/high leptin:** higher than normal probability of having high concentrations of triglycerides and leptin and low HDL-cholesterol levels

**Low leptin/IGF-1/HbA1c:** higher than normal probability of having low levels of leptin, IGF-1 and HbA1c, medium to high levels of ferritin.

**Table 1:** Item-response probabilities in the identified biomarker statuses in the IDEFICS/I.Family cohort (n = 1295). The numbers provide the probabilities of children having low (≤ P15), medium (> P15 and ≤ P85) or high levels (> P85) of the eight biological markers, respectively, in the four latent groups reflecting children with distinct biomarker status. Item-response probabilities were constrained to be equal at baseline and follow-up.

		Normal		Inflammation		Dyslipidemia & high leptin		Low Leptin/IGF-1/HbA1c	
		Prob	95% CI	Prob	95% CI	Prob	95% CI	Prob	95% CI
**Low**	HbA 1c	15.5	(9.9;19.9)	16.4	(10.2;22.5)	17.6	(11.4;24.3)	25.0	(18.4;32.1)
	Triglycerides*	NA		NA		NA		NA	
	HDL-Chol	2.7	(0.0;7.4)	8.2	(0.0;19.1)	59.5	(44.4;76.5)	8.0	(2.0;15.8)
	CRP	27.8	(20.1;35.3)	0.9	(0.0;4.6)	12.6	(6.1;20.3)	24.5	(12.9;36.3)
	Interleukin-6	28.1	(20.8;35.4)	3.9	(0.0;8.9)	6.7	(2.3;12.4)	27.4	(18.0;37.2)
	Leptin	12.2	(6.6; 17.8)	8.9	(0.0;7.0)	6.4	(2.4;10.7)	33.7	(23.7;44.7)
	IGF- 1	0.4	(0.0;6.2)	13.6	(4.4;25.4)	10.0	(3.7;17.0)	50.6	(28.5;87.7)
	Ferritin	27.6	(20.8;35.5)	4.8	(0.0;9.8)	17.5	(11.5;24.4)	12.3	(5.8;20.3)
**Medium**	HbA 1c	74.6	(69.5;79.8)	65.6	(59.4;71.3)	69.8	(62.9;76.7)	70.4	(63.9;76.9)
	Triglycerides	93.5	(89.9;96.8)	96.3	(88.3;100)	49.9	(32.4;64.0)	96.8	(92.0;100)
	HDL-Chol	75.4	(68.7;81.3)	77.0	(68.3;87.0)	40.0	(23.2;54.8)	71.1	(61.1;81.8)
	CRP	71.2	(64.3;77.7)	74.7	(65.3;82.3)	68.8	(62.2;76.0)	67.0	(58.3;74.9)
	Interleukin 6	69.4	(62.9;75.4)	69.2	(59.7;76.6)	77.0	(70.0;85.4)	62.8	(55.2;70.1)
	Leptin	82.3	(77.2;87.2)	70.9	(60.1;79.0)	63.0	(53.5;72.6)	63.8	(53.3;73.2)
	IGF-1	81.7	(74.7;87.6)	72.7	(62.5;80.9)	67.2	(59.0;75.2)	49.4	(12.2;71.4)
	Ferritin	69.1	(62.3;74.8)	72.4	(62.7;74.8)	63.4	(55.2;71.4)	67.8	(58.3;75.9)
**High**	HbA 1c	10.4	(6.9;14.4)	18.0	(12.3;25.1)	12.6	(7.4;18.0)	4.6	(1.1;8.6)
	Triglycerides	6.5	(3.2;10.1)	3.7	(0.0;11.7)	50.1	(36.0;67.6)	3.2	(0.0;8.0)
	HDL-Chol	21.9	(15.4;29.2)	14.9	(5.5;24.9)	0.5	(0.0;2.5)	20.8	(9.5;32.8)
	CRP	1.1	(0.0;4.9)	24.3	(16.4;34.0)	18.6	(9.2;26.3)	8.5	(1.9;16.3)
	Interleukin 6	2.4	(0.0;6.6)	27.0	(18.2;37.4)	16.3	(6.6;23.7)	9.7	(1.9;18.2)
	Leptin	5.5	(2.5;9.2)	20.2	(10.7;34.5)	30.6	(19.8;40.7)	2.5	(0.0; 7.2)
	IGF- 1	17.8	(11.9;24.8)	13.7	(4.7;23.6)	22.7	(15.7;30.3)	0.0	(0.0;0.0)
	Ferritin	3.3	(0.0;8.0)	22.8	(14.9;33.3)	19.2	(9.6;27.6)	19.9	(11.8;28.9)

P15, P85: age- and sex specific percentiles (for triglycerides only sex-specific) calculated based on the largest sample available

Prob: probability

95% CI: 95% confidence interval; bias-corrected bootstrap confidence intervals estimated using 5000 replicates (sample size: N = 1295)

CRP, high sensitivity C-reactive protein; IGF-1, insulin like growth factor 1

*Note: 50.7% of triglyceride values fell below the detection limit at baseline. For this reason, triglycerides where only classified as either medium or high

In a second step, multivariate mixed effects models were used to assess the age-dependent associations between lifestyle factors, non-modifiable risk factors and the transformed probabilities of being in the status”inflammation”, “dyslipidemia/high leptin” or “low leptin/IGF-1/HbA1c”, respectively, considering the two time points simultaneously (details are provided in the S1 File). The ‘normal’ status served as reference category. The models were run separately for each exposure. All models included an interaction term between the exposure and age as well as adjustment variables which were selected based on the directed acyclic graph (DAG) presented in the S1 File; the corresponding adjustment sets are displayed in the S1 File. Results are presented as OR with corresponding 95% confidence intervals (CI).

## Results

Baseline characteristics as well as distributions of exposures among the four identified clinical biomarker statuses are depicted in Table 2. The mean age at T0 was 6.1 years (SD: 1.7) and the sexes were almost balanced (51.5% males). The mean BMI z-score of the child, but also maternal BMI were highest in children in the “dyslipidemia/high leptin” status while being lowest in the “low leptin/IGF-1/HbA1c” status. The highest percentage of children from parents with a high ISCED level was in the “low leptin/IGF-1/HbA1c” status; percentages of familial hypertension, diabetes, and dyslipidemia were lowest. Differences among the groups with regard to lifestyle factors such as fruit and vegetable consumption or media in the bedroom were rather small. Concentrations of individual clinical biomarkers across different biomarker statuses as well as for boys and girls are depicted in the S1 File.

**Table 2:** Description of the IDEFICS/I.Family study population: Means and 95% confidence intervals for continuous variables and numbers and percentages for categorical variables in the total sample as well as in the different latent biomarker groups at baseline (imputed dataset).

	All (N = 1295)		Normal (N = 454)		Inflammation (N = 478)		Dyslipidemia & high leptin (N = 149)		Low Leptin/IGF-1/HbA1c (N = 214)	
	Mean	SD	Mean	SD	Mean	SD	Mean	SD	Mean	SD
**Age [years]**	6.3	1.7	6.4	1.8	6.3	1.7	6.3	1.8	6.1	1.8
**BMI z-score by Cole (2012)**	0.2	1.1	0.1	0.9	0.3	1.1	0.7	1.3	-0.4	0.9
**Maternal BMI [kg/m** ^**2**^ **]**	23.8	4.3	23.7	4.2	24.0	4.5	24.6	4.6	23.0	4.0
**Breast-feeding duration [month]**	7.6	6.2	7.5	5.9	7.3	6.6	8.1	6.4	8.1	6.0
**Birth weight [gram]**	3408.9	569.2	3447.9	575.0	3356.4	538.6	3403.5	574.5	3446.9	612.8
**Fruit/vegetable frequency [times/day]**	2.8	1.7	2.8	1.7	2.8	1.8	2.9	1.6	2.9	1.7
**Preserved food freq [times/day]**	1.0	0.8	1.0	0.8	0.9	0.8	1.0	0.7	1.1	0.9
**Well-being score**	40.4	4.5	40.6	4.6	40.2	4.5	40.2	4.7	40.5	4.3
	**N**	**%**	**N**	**%**	**N**	**%**	**N**	**%**	**N**	**%**
**Males**	667	51.5	228	50.2	245	51.3	79	53.0	115	53.7
**Females**	628	48.5	226	49.8	233	48.7	70	47.0	99	46.3
**Low/medium ISCED**	567	43.8	192	42.3	220	46.0	72	48.3	83	38.8
**High ISCED**	728	56.2	262	57.7	258	54.0	77	51.7	131	61.2
**Member in sports club**	748	57.8	268	59.0	271	56.7	83	55.7	126	58.9
**Not member in sports club**	547	42.2	186	41.0	207	43.3	66	44.3	88	41.1
**0 media in bedroom**	874	67.5	297	65.4	323	67.6	102	68.5	152	71.0
**>0 media in bedroom**	421	32.5	157	34.6	155	32.4	47	31.5	62	29.0
**No familial hypertension**	1073	82.9	374	82.4	394	82.4	116	77.9	189	88.3
**Familial hypertension**	222	17.1	80	17.6	84	17.6	33	22.2	25	11.7
**No familial diabetes**	1248	96.4	432	95.2	462	96.7	142	95.3	212	99.1
**Familial diabetes**	47	3.6	22	4.9	16	3.4	7	4.7	2	0.9
**No familial dyslipidemia**	1172	90.5	410	90.3	436	91.2	131	87.9	195	91.1
**Familial dyslipidemia**	123	9.5	44	9.7	42	8.8	18	12.1	19	8.9
**Italy**	79	6.1	26	5.7	35	7.3	7	4.7	11	5.1
**Estonia**	304	23.5	139	30.6	77	16.1	35	23.5	53	24.8
**Cyprus**	35	2.7	3	0.7	19	4.0	3	2.0	10	4.7
**Belgium**	85	6.6	24	5.3	32	6.7	5	3.4	24	11.2
**Sweden**	205	15.8	80	17.6	69	14.4	19	12.8	37	17.3
**Germany**	273	21.1	82	18.1	104	21.8	48	32.2	39	18.2
**Hungary**	148	11.4	59	13.0	52	10.9	22	14.8	15	7.0
**Spain**	166	12.8	41	9.0	90	18.8	10	6.7	25	11.7

LCL: lower 95% confidence limit

UCL: upper 95% confidence limit

**Fig 2:**
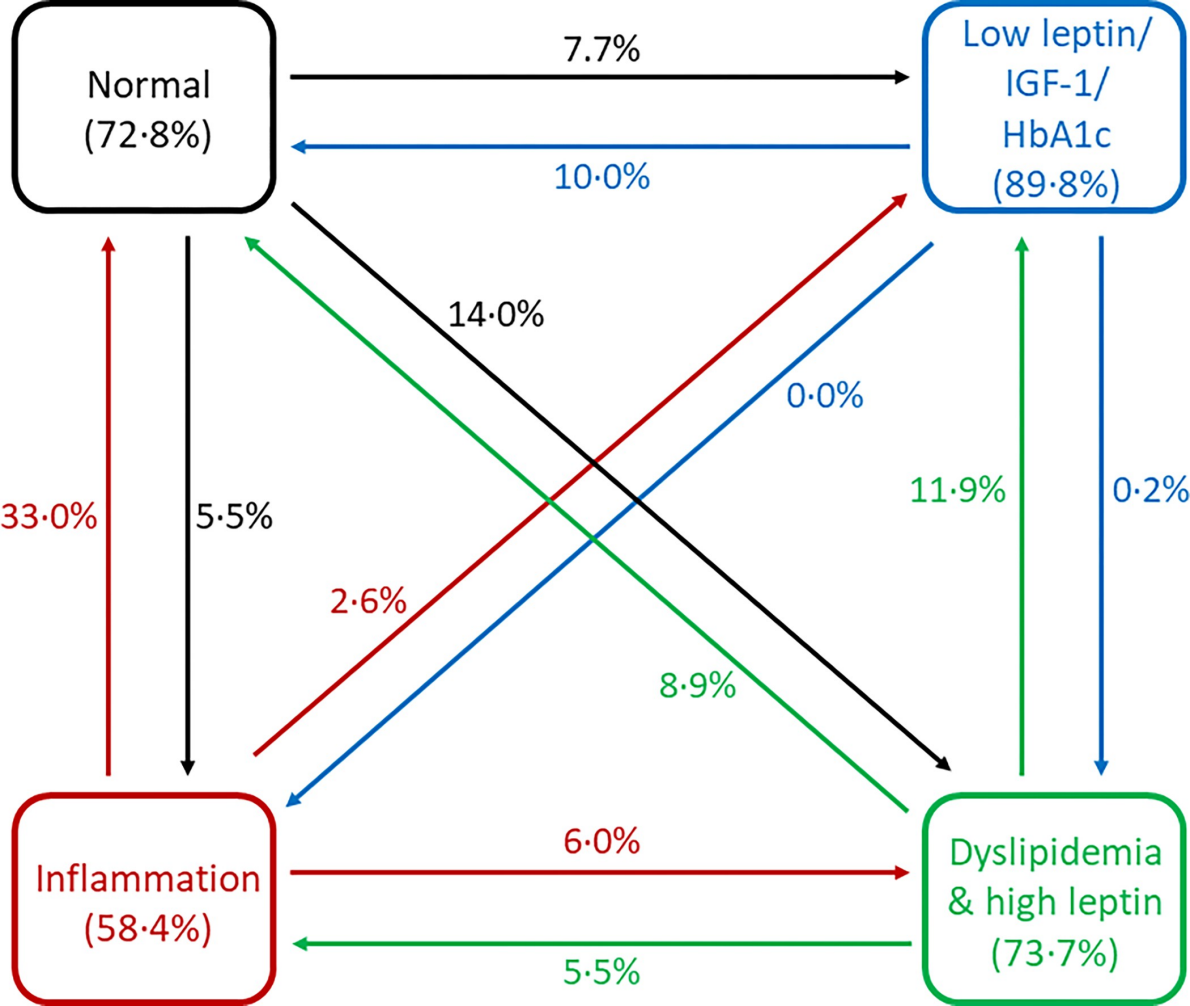
Transition probabilities of children across biomarker statuses over time in the IDEFICS/I.Family cohort (n = 1295). Presented are transition probabilities to stay in the same biomarker status from baseline (2007/08) to follow-up (2013/14) (rectangles) and probabilities to move to another biomarker status at follow-up (arrows).

Prevalence of the four clinical biomarker statuses at T0 and FU are presented in Table 3. At T0, the “Inflammation” status (36%) followed by the “normal” status (32%) showed the highest prevalence, while at FU the prevalence in the “normal” status (37%) was highest. Transition probabilities to stay in the same or move from one to another status are displayed in Fig 2 and the S1 File. Children in the “low leptin/IGF-1/HbA1c” status had the highest probability of staying in the same status at FU (89.8%) while the “inflammation” status revealed the least stability with only 58.4% of children remaining in that status at both waves. Apart from staying in the same status, children in the “normal” status at T0 had a high probability (14%) of moving to the “dyslipidemia/high leptin” status at FU. Children in the “inflammation” status at T0 (33.0%) and in the “low leptin/IGF-1/HbA1c” status (10.0%) showed the highest transition probability for moving to the “normal” status at FU.

**Table 3:** Prevalence of biomarker statuses at baseline (T0) and follow-up (FU) in the IDEFICS/I.Family cohort (n = 1295) estimated based on latent transition analysis (probabilities for group memberships at T0 and FU and 95% confidence intervals).

	Normal	Inflammation	Dyslipidemia & high leptin	Low Leptin /IGF-1/HbA1c
	Prob (95% CI)	Prob (95% CI)	Prob (95% CI)	Prob (95% CI)
**T0**	0.32 (0.30; 0.33)	0.36 (0.34; 0.38)	0.14 (0.13; 0.15)	0.19 (0.17; 0.20)
**FU**	0.37 (0.35; 0.39)	0.23 (0.22; 0.25)	0.17 (0.16; 0.19)	0.22 (0.21; 0.24)

Prob: probability for group membership

95% CI: 95% confidence interval; confidence intervals calculated based on sample post probabilities

The odds ratios (OR), 95% confidence intervals (CI) and p-values for the adjusted associations between our exposures and the biomarker statuses (reference status: normal) as well as interactions with age are presented in Table 4. Children of parents with a low/medium ISCED level showed an increased risk of being in the “inflammation” (1.55 [1.09;2.19]; OR and CI) or “dyslipidemia/high leptin” status (1.49 [1.01;2.21]) at age 8 with the effect increasing with age. Children of families with familial hypertension or diabetes have a decreased risk of being in the “low leptin/IGF-1/HbA1c” status as compared to the “normal” status. Unexpectedly, a longer total breast-feeding duration was associated with a higher risk of being in the “inflammation” (1.04 [1.01;1.07]), “dyslipidemia/high leptin” (1.07 [1.03;1.11]) as well as “low leptin/IGF-1/HbA1c” status (1.06 [1.02;1.11]) as compared to the “normal” status.

**Table 4:** Odds Ratios and 95% confidence intervals for the associations of modifiable and non-modifiable factors with the risk of showing the three distinct biomarker statuses in the IDEFICS/I.Family cohort (n = 1295); reference status: Normal (status 1); back transformed results of the multivariate mixed effects models.

	Inflammation (ref: Normal)				Dyslipidemia & high leptin (ref: Normal)				Low Leptin/IGF-1/ HbA1c (ref: Normal)			
Exposure	OR	LCL	UCL	p-value	OR	LCL	UCL	p-value	OR	LCL	UCL	p-value
**Age**	0.91	0.82	1.02	0.098	**0.86**	**0.76**	**0.98**	**0.019**	**0.80**	**0.70**	**0.91**	**0.001**
**Female sex (ref: male)**	0.84	0.58	1.21	0.346	0.85	0.56	1.30	0.453	0.96	0.61	1.51	0.859
Female sex*age	1.04	0.97	1.12	0.227	1.02	0.95	1.10	0.508	1.07	0.99	1.15	0.071
**ISCED of parents (low/medium; ref: high)**	**1.55**	**1.09**	**2.19**	**0.014**	**1.49**	**1.01**	**2.21**	**0.044**	0.90	0.59	1.37	0.631
ISCED of parents*age	**1.09**	**1.01**	**1.17**	**0.027**	**1.13**	**1.05**	**1.22**	**0.001**	1.07	0.99	1.15	0.083
**Familial hypertension (yes; ref: no)**	1.14	0.69	1.89	0.604	1.16	0.65	2.06	0.619	**0.53**	**0.29**	**0.99**	**0.045**
Familial hypertension*age	1.03	0.94	1.13	0.538	1.03	0.93	1.13	0.616	1.02	0.92	1.13	0.688
**Familial diabetes (yes; ref: no)**	0.73	0.27	1.96	0.535	0.63	0.20	1.94	0.418	**0.26**	**0.08**	**0.86**	**0.027**
Familial diabetes*age	1.07	0.89	1.30	0.468	1.07	0.88	1.30	0.490	1.06	0.87	1.29	0.556
**Familial dyslipidemia (yes; ref: no)**	0.87	0.46	1.63	0.659	1.33	0.64	2.74	0.443	0.74	0.34	1.62	0.453
Familial dyslipidemia*age	1.03	0.91	1.16	0.672	1.09	0.96	1.23	0.193	1.07	0.94	1.22	0.295
**Maternal BMI**	1.04	1.00	1.09	0.067	1.05	0.99	1.10	0.087	**0.93**	**0.88**	**0.98**	**0.011**
Maternal BMI*age	1.00	1.00	1.01	0.350	**1.01**	**1.00**	**1.02**	**0.032**	1.00	0.99	1.01	0.855
**Birth weight (1 unit~100g)**	0.99	0.96	1.03	0.687	1.00	0.96	1.03	0.806	1.01	0.97	1.05	0.675
Birth weight*age	1.00	0.99	1.01	0.775	1.00	1.00	1.01	0.279	1.00	0.99	1.01	0.704
**Breast-feeding duration (1 unit~1 month)**	**1.04**	**1.01**	**1.07**	**0.013**	**1.07**	**1.03**	**1.11**	**0.000**	**1.06**	**1.02**	**1.11**	**0.003**
Breast-feeding*age	1.00	0.99	1.01	0.871	1.00	0.99	1.00	0.390	1.00	0.99	1.00	0.392
**Fruit/vegetable consumption**	1.09	0.99	1.20	0.086	1.10	0.99	1.21	0.065	1.09	0.99	1.20	0.084
Fruit/vegetable consumption*age	1.00	0.98	1.02	0.653	**0.97**	**0.96**	**0.99**	**0.010**	0.99	0.97	1.01	0.267
**Processed food consumption**	1.11	0.91	1.37	0.298	1.03	0.83	1.28	0.793	1.16	0.92	1.45	0.211
Processed food consumption*age	1.04	0.99	1.09	0.091	1.01	0.96	1.07	0.575	1.04	0.99	1.09	0.156
**Number of media (≥1; ref: 0)**	**1.46**	**1.01**	**2.11**	**0.046**	1.15	0.75	1.75	0.525	0.98	0.64	1.50	0.937
Number of media*age	**1.14**	**1.02**	**1.28**	**0.018**	**1.28**	**1.13**	**1.44**	**< .0001**	1.10	0.98	1.24	0.100
**Well-being score (1 unit~10 points)**	0.79	0.57	1.10	0.164	0.87	0.61	1.24	0.443	0.90	0.62	1.31	0.590
Well-being score*age	0.97	0.89	1.06	0.488	**0.90**	**0.82**	**0.98**	**0.012**	0.97	0.89	1.06	0.560
**Sports club (yes; ref: no)**	**0.72**	**0.53**	**0.98**	**0.036**	**0.60**	**0.44**	**0.83**	**0.002**	0.96	0.68	1.35	0.803
Sports club*age	0.98	0.90	1.06	0.615	0.95	0.88	1.03	0.247	1.00	0.92	1.09	0.974
**Entered puberty (yes; ref: no)**	0.99	0.25	3.90	0.984	1.63	0.39	6.80	0.500	0.22	0.05	1.01	0.052
Entered puberty*age	0.94	0.73	1.21	0.634	0.96	0.74	1.25	0.752	1.15	0.88	1.52	0.305
**BMI z-score of child**	1.12	0.96	1.30	0.144	**1.54**	**1.31**	**1.81**	**< .0001**	**0.32**	**0.27**	**0.38**	**< .0001**
BMI z-score*age	**1.09**	**1.06**	**1.13**	**< .0001**	**1.13**	**1.09**	**1.17**	**< .0001**	**1.04**	**1.00**	**1.08**	**0.028**

Continuous variables were centred (and rescaled) to: 8 years of age, 3500 g birth weight (1 unit~ 100g), 6 months of breast-feeding, maternal BMI of 23 kg/m^2^, eating processed food 0 times/day, eating fruits and vegetables 5 times/day, well-being score of 40 (1 unit~10 points).

Statistically significant results are shown in bold.

Exposures were adjusted using the confounders identified based on the DAG. The adjustment sets for all exposures are given in the S1 File. All models were adjusted for age, sex and country of residence. Estimates were corrected for multiple imputation.

ISCED: International Standard Classification of Education; LCL: lower 95% confidence limit; OR: Odds Ratio; ref: reference category; UCL: upper 95% confidence limit

Out of the lifestyle factors, a higher number of media in bedroom increased the risk of being in the “inflammation” status (1.46 [1.01;2.11] at age 8; 1.14 [1.02;1.28] for interaction with age, i.e. resulting in an OR of 1.46*1.14 = 1.66 at age 9, 1.46*1.14*1.14 = 1.90 at age 10 and so forth) and “dyslipidemia/high leptin” status (1.15 [0.75;1.75]; 1.28 [1.13;1.44] for interaction with age) with the effects markedly increasing with age. Membership in a sports club decreased the risk of being in the “inflammation” (0.72 [0.53;0.98]) and “dyslipidemia/high leptin” (0.60 [0.44;0.83]) status. Although the well-being score at age 8 (main effect) was not associated with the “dyslipidemia/high leptin” status, the age interaction was statistically significant (p = 0.012) revealing that a higher well-being score is negatively associated with the “dyslipidemia/high leptin” status at older ages. Children with a higher BMI z-score had a higher risk of being in the “inflammation” and “dyslipidemia/high leptin” status and a lower chance of being in the “low leptin/IGF-1/HbA1c” status at age 8 with the effects again increasing with age. The dietary variables considered showed only small or no associations with the different biomarker statuses. Although the effect estimates for entering puberty and the puberty-age-interaction did not reach statistical significance, estimates point to the direction of pubertal children being less likely to be classified as “low leptin/IGF-1/HbA1c” (0.22 [0.05;1.01]). Unadjusted effect estimates are shown in the S1 File. The estimates were in general similar to those obtained from the confounder-adjusted models.

## Discussion

In our study, we identified four distinct biomarker statuses based on comprehensive data and repeatedly collected blood samples of a large European children cohort including: “normal”, “low leptin/IGF-1/HbA1c”, “inflammation” and “dyslipidemia/high leptin”.

### Characterization of the different biomarker statuses

The “low leptin/IGF-1/HbA1c” status and the “normal” status were both characterized by high HDL-cholesterol, and low triglycerides and inflammatory markers. However, the “low leptin/IGF-1/HbA1c” status was additionally characterized by low levels of leptin, IGF-1 and HbA1c, and medium to high serum ferritin. Children in the “low leptin/IGF-1/HbA1c” group were slightly younger and thinner than the other children, which may have contributed to lower leptin and lower IGF-1 levels. Leptin belongs to the class of adipokines. It is mainly secreted by adipose tissue to regulate energy balance, body mass and reproductive function via multiple metabolic pathways including proinflammatory immune response [24]. IGF-1 is traditionally used in children to diagnose growth hormone deficiency, but IGF-1 concentrations may also change in other metabolic states such as obesity [9]. IGF-1 concentrations have been previously reported to increase with age up to puberty during childhood and to be elevated in children with overweight/obesity [25]. In addition, higher IGF-1 concentrations at age 12 months predicted higher length, weight and head circumference at 18 months in toddlers from Tansania [19].

The biomarker status “dyslipidemia/high leptin” was characterized by higher triglycerides, leptin, IGF-1 and lower HDL-cholesterol concentrations as compared to the “normal” status. In a recent study of Norwegian children and adolescents, blood concentrations of leptin, IGF-1 and triglycerides were also positively associated with each-other and negatively associated with HDL-cholesterol concentrations, and were particularly elevated in children with overweight/obesity [26]. In a US cohort increasing leptin trajectories during childhood were linked to higher risk of obesity [16, 17]. In line with previous studies we found that the mean BMI z-scores were highest in children in the “dyslipidemia/ high leptin” group. This finding may imply that already in children with overweight/obesity next to dyslipidemia also increased leptin synthesis and secretion and pre-stages of leptin resistance may exist. A consequence may be disturbed leptin signaling and reduced satiety in children with overweight/obesity [27]. In addition, obesity may result in increased IGF-1 levels. Previous studies have reported that growth hormone secretion is reduced in children with obesity while IGF-1 is typically mildly increased, and IGF-1 may be one factor involved in the accelerated growth of pre-pubertal children with obesity [9, 28]. Leptin, on the other hand, is a key sensor of energy availability in the body. Previous experimental studies showed that IGF-1 reduces leptin expression in white adipose tissues [29, 30]. However, it has also been observed that IGF-1 increased leptin expression in other tissues [31]. Interestingly, it has very recently been shown that administration of leptin to leptin-deficient children and adults was accompanied by an increase in serum IGF-1 [32].

The”inflammation” status was characterized by a higher probability of high levels of CRP, IL-6 and ferritin but also high leptin. Apart from acute stages of inflammation resulting e.g. from infections, elevated levels of CRP and IL-6 are also observed in a condition coined as low-grade inflammation such as obesity. The “inflammation” group in our study was characterized by slightly higher BMI than the “normal” and “low leptin/IGF-1/HbA1c” group but lower BMI as compared to the “dyslipidemia/high leptin” group. High serum ferritin levels may reflect inflammation and cell damage and/or iron overload [33]. Therefore, it is common to interpret serum ferritin concentrations in parallel with serum CRP levels [34]. As we also observed high CRP levels in the “inflammation” group, higher ferritin may reflect its inflammatory function in our study. Leptin may be a key link between the neuroendocrine and the immune system and has been suggested to be a pro-inflammatory factor itself, e.g. by leptin-signaling stimulated cytokine production [35]. To sum up, two of the biomarker statuses that we observed were characterized by higher concentrations of classical clinical biomarkers such as CRP and lipid profiles but also by high leptin and high IGF-1. This finding supports the hypothesis that the different pathways of dietary intake, inflammation, lipid metabolism, energy homeostasis, satiety and growth are highly interlinked.

There is a complex interplay of nutrition, inflammation and chronic disease risk. Previous studies reported that fast food dietary patterns in contrast to vegetable-based patterns were linked to higher concentrations of inflammatory biomarkers, and a vegan diet was reported to reduce CRP concentrations [36, 37]. Possible biological mechanisms include diet-induced changes in gut microbiota composition [38]. High fiber intake induces production of short-chain fatty acids by gut microbiota, and has beneficial effects on inflammation, lipid and glucose metabolism, gut barrier integrity and immune function; whereas high animal protein diets cause synthesis of trimethylamine N-oxide, a metabolite linked to cardiovascular disease risk [39]. Inflammatory diets are usually characterized by high intake of meat and consequently heme iron; and have been associated with risk of cancer, cardiovascular diseases and type 2 diabetes in previous epidemiological studies [40–43]. In our children cohort, we previously observed that heme iron intake was a strong predictor of plasma ferritin concentration [44]. Ferritin, itself, has been found as one mediating biomarker for the red meat–type 2 diabetes—association [45, 46]. Iron overload causes oxidative stress and cell damage, whereas iron deficiency reduces immune function, and both mechanisms are involved in the development of chronic diseases [41]. Fat quality of the diet is another important factor that can alter inflammatory and metabolic state. Particularly fast-food diets are high in saturated, trans-unsaturated and omega-6 polyunsaturated fatty acids, and low in omega-3 polyunsaturated fatty acids. This fatty acid pattern shows proinflammatory properties and has been linked to risk of obesity, dyslipidemia and metabolic syndrome also in children [47–49].

### Transitions of biomarker status over time and determinants of biomarker status

Our results showed that children classified as “low leptin/IGF-1/HbA1c” had the highest probability to stay in that group whereas e.g. children from the “normal group” had a non-negligible risk of drifting into the “dyslipidemia/high leptin” group. Our study suggests that particularly children characterized by lower concentrations of leptin, IGF-1 and HbA1c are likely to maintain this status in adolescence. Determinants of the “low leptin/IGF-1/HbA1c” biomarker status included younger age, lower BMI, and prolonged breast-feeding duration; but particularly several parental characteristics revealed strong association such as no family history of diabetes and hypertension, and lower maternal BMI. Thus, the parents may play a key role for individual biomarker status of their children due to genetic factors and/or role modeling function.

The “inflammation” group was the least stable group over time. For some children their inflammatory status may have been more acute and flexible whereas for others it may be already a chronic stage of inflammation, e.g. through pathophysiological pathways of obesity. This is in line with a previous study that reported lowest CRP levels for overweight children that transitioned to normal weight, and highest CRP concentrations for children with manifest obesity [18]. It has been previously suggested that a positive feedback loop exists, so that local inflammation in adipose tissue enhances altered immune response in obesity and vice versa. This process is also mediated via pro-inflammatory markers secreted by adipose tissue such as leptin [50]. Children in the “inflammation” and “dyslipidemia/high leptin” status were particularly characterized by higher BMI, higher number of medias in the bedroom, lower parental education and not being a sports club member in our study. Media time has been linked with sedentary behavior and poor diet quality including an increased intake of foods with high amounts of sugar and fat, as well as sugar-sweetened beverages [51]. Our study further underscores the previously identified prevention target to reduce media time and increase physical activity in children [52]. In contrast to results from previous studies [17, 53], entering puberty was not statistically significantly associated with any biomarker status in our study. However, effect estimates were quite large and pointed to the expected directions, though with wide confidence intervals. Our study may have lacked statistical power as all children were pre-pubertal at T0 and only 45.1% had entered puberty at FU. In a previous investigation, pubertal children showed higher total and LDL-cholesterol concentrations, and by tendency higher leptin concentrations than their counterparts [17]. In addition, it is known that IGF-1 levels rise up to and during puberty [53]. This is in line with the (non-significant) higher probability of pubertal children being in the “dyslipidemia & high leptin” status and lower probability of being in the “low leptin/IGF-1/HbA1c” status in our study. Perng et al. 2019 [17] further reported sex-specific differences for adipokines such as a steeper increase for leptin in girls, and larger decrease of adiponectin in boys during adolescence. Biological sex was not related to any of the four biomarker statuses in our study.

Longer total breast-feeding duration increased the likelihood for being in the “inflammation”, “dyslipidemia & high leptin” and “low leptin/IGF-1/HbA1c” status compared to the “normal” status in our study. Recent meta-analyses reported that breast-feeding reduced risk of obesity and metabolic syndrome in children [54, 55]; and thus, breast-feeding is likely to influence biomarker levels in children. As we did not expect a positive association of breast-feeding with the “inflammation” and “dyslipidemia/high leptin” biomarker status, we conducted sensitivity analyses (see S1 File) using (1) exclusive breast-feeding as well as (2) a binary indicator for ‘at least 6 months exclusive breast feeding’ (vs ‘less than 6 months’) instead of total breast-feeding duration (including breast-feeding combinations e.g. with formula milk). However, results still pointed to the unexpected direction. Despite having rich data and using a DAG to derive an appropriate adjustment set, we cannot preclude unmeasured confounding.

### Strengths and limitations

Our study has the unique strength of being based on a large, well-phenotyped cohort of European children. This enabled us to study changes in biomarker status in the transition period from childhood to adolescence while considering a comprehensive set of potential determinants of biomarker status. Comparable data is particularly rare in children. All examinations were based on quality control procedures and followed a highly standardized protocol. Sophisticated statistical methods were used to reduce the dimensionality of the data and to assess the clustering and progression of biological markers over time. We categorized our biomarkers based on age- and sex-specific percentiles as biomarkers often show a highly skewed distribution making other clustering algorithms either difficult to apply (e.g. Gaussian mixture models rely on the assumption of multivariate normal distribution) or difficult to interpret (if biomarkers are transformed beforehand). Nevertheless, a larger number of categories for biomarker classification would have been desirable. Our LTA model did not converge when using more than three biomarker categories and was also less stable when using the more common 10^th^ and 90^th^ percentiles for classification due to smaller numbers of observations in the extreme classes. This corroborated our decision to use three categories based on the 15^th^ / 85^th^ percentiles. We included only children participating in the baseline as well as second follow-up examination of the IDEFICS/I.Family cohort. Drop-out over time was high; only 46% of the children participating in the baseline and first follow-up examination participated also in the second follow-up examination [56]. A previous analysis showed attrition in IDEFICS/I.Family to be associated with a lower parental education, parent’s migrant background, higher weight status of children, lower children’s study compliance as well as older age. Also, when comparing our analysis sample with the overall IDEFICS/I.Family cohort (described in Ahrens et al. [20, 57]), our sample shows a selection towards a healthier population of children with slightly lower mean BMI z-score and better educated parents.

We preselected eight biomarkers reflecting different biological pathways based on availability of measurements for most children. However, a different selection of clinical biomarkers may have led to different biomarker statuses. Exploratory approaches such as metabolomics measurements may further highlight unexpected metabolites and pathways shifting from childhood to adolescence [58]. We also suggest that future studies should include more than two time points with shorter time resolution to better detangle development of biomarker levels during childhood. Our dietary indicators revealed no or only small associations with the biomarker statuses. Especially dietary proxy- and self-reports may be prone to misreporting and social desirability bias which may have contributed to this (unexpected) null finding. BMI (z-score) was used as simple indicator for overweight and obesity, which is common practice. However, BMI does not necessarily reflect body fat and particularly the fat distribution in children. Bedroom devices were used as a rather simple proxy for media time of children. However, we previously showed that children from the same cohort with bedroom devices are less likely to meet the screen time recommendations [59]. Lastly, we used data from a prospective cohort study, which is of observational design. Therefore, we can report associations of biological markers. Causality of biological pathways, however, should be checked in experimental study designs.

## Conclusions

We found that particularly children characterized in the biomarker status with low concentrations of leptin, IGF-1 and HbA1c, in addition to low levels of triglycerides and CRP and high concentrations of HDL-cholesterol in early childhood were most likely to retain these biomarker levels over years. These children showed a lower body mass index (BMI), were less likely to have a family history of diabetes or hypertension and their parents had a high educational level and lower maternal BMI. In contrast, children with obesity and/or a family history of diabetes/hypertension are at risk of adverse development of biomarker profiles from childhood into adolescence. Our study highlights the role of the parents for child health and the need to reduce health inequalities for families. Important prevention goals in children are to retain a normal body weight and to promote an active lifestyle.

## Supporting information

S1 File Floegel et al. (2024). Transition of clinical biomarker status from childhood into adolescence–a prospective study in children from eight European countries.
(DOCX)
