# Supplementary material for: Transition of clinical biomarker status from childhood into adolescence–A prospective study in children from eight European countries
Source: PLoS One. 2025 Feb 3;20(2):e0311180. doi: 10.1371/journal.pone.0311180 (PMC11790143; doi:10.1371/journal.pone.0311180)
Supplement: S1 File — Transition of clinical biomarker status from childhood into adolescence–a prospective study in children from eight European countries. (DOCX) [file pone.0311180.s001.docx]

**Supplementary material**

Floegel et al. (2024). Transition of clinical biomarker status from childhood into adolescence – a prospective study in children from eight European countries.

**Table of contents**

[**S1 File:** Determinants of biomarker status 2](#_Toc176958898)

[**S2 Table:** Classification of biomarkers 5](#_Toc176958899)

[**S3 File:** Details on the latent transition analysis 7](#_Toc176958900)

[**S4 Table:** Model selection criteria for the latent transition analysis that was performed assuming 2 up to 7 latent statuses 8](#_Toc176958901)

[**S5 File:** Description of the multivariate mixed effects models and multiple imputation 9](#_Toc176958902)

[**S6 Fig:** Directed acyclic graph (DAG) depicting the causal relations assumed among determinants of biomarker status 11](#_Toc176958903)

[**S7 Table:** Exposures considered in the analyses and minimal adjustment sets 12](#_Toc176958904)

[**S8 Table:** Median, means and standard deviations for the concentrations of different biomarkers in the total analysis sample, in the four derived latent biomarker groups as well as by sex 14](#_Toc176958905)

[**S9 Table:** Transition probabilities (and 95% confidence intervals) from T0 to FU 15](#_Toc176958906)

[**S10 Table:** Odds Ratios and 95% confidence intervals for the unadjusted associations 16](#_Toc176958907)

[**S11 Table:** Sensitivity analyses for breast feeding duration 18](#_Toc176958908)

[**References** 19](#_Toc176958909)

# **S1 File:** Determinants of biomarker status

***Lifestyle factors***

If not otherwise stated, variables are obtained at T0 and during FU based on proxy-reported questionnaires completed by parents or guardians. At FU children ≥12 years reported for themselves.

Dietary variables

A variable indicating daily consumption frequencies of fruits and vegetables was calculated summing the reported frequencies of a) fresh fruits without sugar added, b) fresh fruits with sugar added, c) cooked vegetables, d) legumes, and e) raw vegetables. Analogously a variable indicating daily consumption frequencies of processed foods was calculated as the sum of a) cold cuts and preserved, meat products, b) snacks like savory pastries and fritters, and c) hamburger, hot dog, kebab, wrap, falafel.

Sports club member

A variable indicating whether the child was member in a sports club was used as proxy for physical activity.

Well-being score

Psychosocial well-being was measured with 16 items of four subscales of the “KINDL-R Questionnaire for Measuring Health-Related Quality of Life (HRQoL) in Children and Adolescents” (emotional well-being, self-esteem, family life and relations to friends).^1,2^ At second FU, response categories corresponded to the original 5-point Likert scale (never, seldom, sometimes, often, all the time). At T0 and the first FU the two highest response categories were combined into one category. Therefore, we deviated from the original scoring (1-5 points per item) and assigned 0 points for “Never” and 3 points for both “Often” and “All the time” (at FU) or “Often/All the time” (at T0), respectively (six negatively worded items were coded reversely). Consequently, the score ranged from 0-48 with a higher score indicating a higher well-being.

Media

The number of media devices reported to be in children’s’/teens’ bedroom was used as an indicator for media use.^3^ In order to reduce model complexity, a dummy was used in the multivariate model indicating children with 0 vs ≥ 1 media in the bedroom.

BMI z-score of the child/adolescent

BMI was calculated as weight divided by height squared. Age- and sex-specific BMI z-scores were calculated based on Cole and Lobstein ^4^.

***Non-modifiable risk factors***

Demographics

Age of the children, sex, country of residence as well as the highest educational level of parents according to the International Standard Classification of Education (ISCED)^5^ as indicator for socio-economic status were considered. Educational level was used as dummy variable in the multivariate model in order to reduce model complexity (low/medium vs high ISCED level; original coding: 1=low, 2=medium, 3=high).

Family history of diseases

Parents reported the presence of metabolic disturbances for themselves, their partner as well as children. Three variables were constructed indicating the presence of hypertension, dyslipidemia and type 2 diabetes in any of the family members (yes vs. no).

Early life factors

Birth weight (g) and total breast-feeding duration (months; including breast-feeding combinations) were reported by mothers either in T0 or first FU wave.

Maternal body mass index (BMI)

Maternal BMI at T0 was calculated as weight (kg) divided by height (m) squared where weight/height were self-reported. If maternal BMI was missing at T0, the first FU value was used.

Pubertal status

Only at FU, pubertal status (yes vs. no; yes if menarche had already occurred in girls or if voice alterations had already started or were completed in boys) was self-reported by children 8 years and older based on questions adapted from Carskadon and Acebo^6^.

# **S2 Table:** Classification of biomarkers

For the eight selected biomarkers, the 15th, and 85th age group- and sex-specific percentiles were calculated using the largest sample available out of the total IDEFICS/I.Family cohort. Three age-groups were chosen (3-<6 years, 6-<12 years and 12-≤15 years) in order to maintain a sufficient sample size for the percentile calculation. Percentiles were calculated separately for the two examination waves as biomarker distributions differed which may be mainly explained by the use of different laboratory assessment methods. All biomarkers were classified in 3 categories according to the derived percentiles (<15^th^ percentile, 15^th^ to < 85^th^ percentile, ≥85^th^ percentile; see Supplementary material 2) indicating “low”, “medium” and “high” biomarker levels. With regard to triglycerides, 50·7% of the values from the first examination wave fell below the detection limit of 45 mg/dL such that the 15^th^ percentile could not be calculated. Here values were only classified as <85^th^ percentile vs ≥ 85^th^ percentile. The Table below shows the number of children in the analysis sample being classified as low, medium or high with respect to the different biomarkers as well as numbers of missing values.

|  | **Low** | | **Medium** | | **High** | | **Missing values** | |
| --- | --- | --- | --- | --- | --- | --- | --- | --- |
|  | N | % | N | % | N | % | N | % |
| **Hba1c T0** | 201 | 15·7 | 907 | 70·8 | 173 | 13·5 | 14 | 1·08 |
| **Hba1c FU** | 251 | 20·1 | 875 | 70·2 | 121 | 9·7 | 48 | 3·71 |
| **TAG T0^§^** | NA | NA | 1093 | 89·2 | 132 | 10·8 | 70 | 5·41 |
| **TAG FU^§^** | NA | NA | 1114 | 87·1 | 165 | 12·9 | 16 | 1·24 |
| **HDL-c T0** | 180 | 14·7 | 855 | 69·8 | 190 | 15·5 | 70 | 5·41 |
| **HDL-c FU** | 176 | 13·8 | 884 | 69·1 | 219 | 17·1 | 16 | 1·24 |
| **CRP T0** | 149 | 16·0 | 670 | 72·0 | 111 | 11·9 | 365 | 28·19 |
| **CRP FU** | 217 | 18·5 | 821 | 70·0 | 135 | 11·5 | 122 | 9·42 |
| **IL-6 T0** | 152 | 17·2 | 603 | 68·1 | 131 | 14·8 | 409 | 31·58 |
| **IL-6 FU** | 206 | 18·3 | 790 | 70·4 | 127 | 11·3 | 172 | 13·28 |
| **Leptin T0** | 124 | 13·9 | 638 | 71·5 | 130 | 14·6 | 403 | 31·12 |
| **Leptin FU** | 183 | 15·5 | 862 | 73·1 | 135 | 11·4 | 115 | 8·88 |
| **IGF-1 T0** | 169 | 15·1 | 796 | 71·3 | 152 | 13·6 | 178 | 13·75 |
| **IGF-1 FU** | 184 | 15·2 | 862 | 71·0 | 168 | 13·8 | 81 | 6·25 |
| **Ferritin T0** | 153 | 16·4 | 654 | 70·0 | 128 | 13·7 | 360 | 27·80 |
| **Ferritin FU** | 208 | 16·8 | 849 | 68·4 | 185 | 14·9 | 53 | 4·09 |

**S2 Table:** Children with low (≤P15), normal (>P15 and ≤P85), and high (>P85) biomarker levels at T0 and FU (number of children and percentages)

P15, P85: age- and sex specific percentiles (for triglycerides only sex-specific) calculated based on the largest sample available considering the total IDEFICS/I.Family cohort.

^§^ Only two categories were derived because 50·7% of the T0 triglyceride values fell below the detection limit

Note: This table is based on a total of 1295 children providing biomarker data at T0 and FU with a maximum of three missing values at each wave. As percentiles were calculated based on the largest sample available out of the total cohort, not exactly 15% of children fall into the low and high categories, respectively.

T0: baseline, FU: follow-up, HbA1c: glycated hemoglobin A1c, TAG: triacylglycerides, HDL-c: high-densitiy lipoprotein cholesterol, CRP: C-reactive protein, IL-6: interleukin-6, IGF-1: insulin-like growth factor-1

# **S3 File:** Details on the latent transition analysis

The categorized biomarkers (HbA1c, HDL-cholesterol, triglycerides, CRP, IL-6, ferritin, leptin and IGF-1) formed the basis to identify groups of children with similar biomarker status: Latent class analysis (LCA) is a latent variable model that is used to identify underlying (unobserved) subgroups in a population ^7^. Latent transition analysis (LTA) is a longitudinal extension of LCA that allows latent class membership to change over time; in this model, change is quantified by a matrix of transition probabilities between two consecutive time points ^8^. LTA was used in the present analysis to identify groups of children with distinct biomarker status as well as transition probabilities over time (i.e. changes in the assignment to the different latent metabolic groups from T0 to second FU wave). LTA models can handle missing data assuming data to be missing at random such that all children with a maximum of three missing biomarker values at T0 and FU were considered.

The eight categorized biomarkers at T0 and FU were used to estimate probabilities (prevalence) for latent statuses at T0 and FU, probabilities for transitions between latent statuses from T0 to FU as well as item-response probabilities conditional on latent status membership (i.e. probabilities of being in the different biomarker categories in the different latent statuses). The item-response probabilities were restricted to be equal across all times. This ensured to detect the same latent classes at T0 and FU which eases interpretability of model estimates and enhanced model fit.

Bootstrap was performed to estimate confidence intervals for the item-response and transition probabilities based on the original sample size of N=1295 with 5000 replicates using unrestricted random sampling. Starting values were estimated based on the initial sample. Bias-corrected (BC method) 95% confidence intervals were estimated to correct for skewness.

# **S4 Table:** Model selection criteria for the latent transition analysis that was performed assuming 2 up to 7 latent statuses

|  | **2 status model** | **3 status model** | **4 status model** | **5 status model** | **6 status model** | **7 status model** |
| --- | --- | --- | --- | --- | --- | --- |
| **Log-likelihood** | -13418·2 | -13316·1 | -13234·7 | -13168·8 | -13124·8 | -13084·8 |
| **G-squared** | 9098·7 | 8894·4 | 8731·6 | 8599·8 | 8511·8 | 8431·7 |
| **AIC** | 9168·7 | 9006·4 | 8889·6 | 8807·8 | 8773·8 | 8751·7 |
| **BIC** | 9349·5 | 9295·8 | 9297·8 | 9345·1 | 9450·6 | 9578·3 |

AIC: Akaike information criterion

BIC: Bayesian information criterion

# **S5 File:** Description of the multivariate mixed effects models and multiple imputation

Let p_normal_ denote the probability of children being assigned to the normal status and analogously p_inflammation,_ p_dyslipidemia_ and p_low leptin/IGF-1/HbA1c_ the probabilities for the other statuses_._ As these probabilities sum up to 1, our data can be considered as compositional data and were analyzed as such following the approach described in Faes, Molenberghs ^20^. Children’s probabilities of being assigned to the different latent statuses at T0 and FU were transformed using the additive logratio transformation:

- p_inflammation_ is transformed to ln(p_inflammation_/ p_normal_)
- p_dyslipidemia_ is transformed to ln(p_dyslipidemia_/ p_normal_)
- p_low leptin/IGF-1/HbA1c_ is transformed to ln(p_low leptin/IGF-1/HbA1c_ / p_normal_)

The transformed values were then used as outcome variables in a multivariate analysis. This enables the interpretation of results after backtransformation in terms of odds ratios (OR), with the normal status serving as the reference group for all other statuses. Multivariate mixed-effects models were used to assess the age-dependent associations between lifestyle factors, non-modifiable risk factors and the transformed probabilities of being in the ”inflammation”, “dyslipidemia/high leptin” or “low leptin/IGF-1/HbA1c” status, respectively, considering the two time points simultaneously. The model included a random subject-specific intercept and accounted for the correlation of measurements taken from the same child. The factor-analytic covariance structure was chosen for the random effects to allow modeling heterogeneous covariances (when using the preferred unstructured covariance structure, the estimated G matrix was not positive definite). Continuous co-variables were centered before model fit to obtain meaningful model estimates.

For missing covariates, multiple imputation was applied using the fully conditional specification (FCS) discriminant method for categorical variables and the FCS predictive mean matching method for continuous variables with 10 replicates. The FCS method assumes an arbitrary missing data pattern and the existence of a joint distribution for these variables (Brand 1999; Van Buuren 2007). All outcomes and exposures used in the final analyses were included in the multiple imputation procedure. The percentages of missing values ranged from 0% (age, sex) up to 13·6% (fruit/vegetable consumption in T0) depending on the variable considered. The relative efficiency was >95% for all variables indicating good imputation quality.

All analyses were performed using SAS® statistical software version 9.3 (SAS Institute, Inc., Cary, NC, USA). Proc LTA was used to conduct the latent transition analysis, Proc MI for the multiple imputation and Proc MIXED for the multivariate mixed-effects models. Estimates for the multiple imputed datasets were combined using Proc MIANALYZE.

**S6 Fig:** Directed acyclic graph (DAG) depicting the causal relations assumed among determinants of biomarker status

The DAG was build using DAGitty (http://www.dagitty.net/). Some variables were further grouped (e.g. dietary variables like fruit/vegetable consumption and processed food consumption were grouped as “Diet”) to reduce the complexity of the DAG. The DAG further includes one unobserved factor indicated in grey – the genes.

# **S7 Table:** Exposures considered in the analyses and minimal adjustment sets

| **Exposure** | **Adjustment set** |
| --- | --- |
| Age | Sex, country of residence |
| Female sex (ref: male) | Age, country of residence |
| ISCED of parents (low medium; ref: high) | Age, sex, country of residence |
| Family history of diabetes, dyslipidemia or hypertension (yes; ref: no) | Age, sex, country of residence, genes |
| Maternal BMI | Age, sex, country of residence, family history of diseases, genes, ISCED level |
| Birth weight (1 unit~100g) | Age, sex, country of residence, ISCED level, maternal BMI |
| Breast-feeding | Age, sex, birth weight, country of residence, ISCED level, maternal BMI |
| Fruit/vegetable consumption | Age, sex, breast-feeding, country of residence, family history of diseases, genes, ISCED level, maternal BMI, media in bedroom, membership in sports club, pubertal stage, wellbeing score |
| Processed food consumption | Age, sex, breast-feeding, country of residence, family history of diseases, genes, ISCED level, maternal BMI, media in bedroom, membership in sports club, pubertal stage, wellbeing score |
| Number of media (≥1; ref: 0) | Age, sex, country of residence, family history of diseases, ISCED level, maternal BMI, pubertal stage |
| Well-being score (1 unit~10 points) | Age, sex, breast-feeding, country of residence, genes, ISCED level, maternal BMI, media in bedroom, membership in sports club, pubertal stage |
| Sports club (no; ref: yes) | Age, sex, c**ountry of residence**, family history of diseases, ISCED level, maternal BMI, media in bedroom, pubertal stage |
| Entered puberty (yes; ref: no) | Age, sex, country of residence, genes |
| BMI z-score of child | Age, sex, birth weight, breast-feeding, c**ountry of residence**, diet, family history of diseases, genes, maternal BMI, media in bedroom, membership in sports club, pubertal stage, wellbeing score |

Variables listed in grey indicated unobserved factors that we were not able to adjust for.

| **T0 status** | **All (N=1295)** | |  |  | **Normal (N=454)** | | |  | **Inflammation (N=478)** | | |  | **Dyslipidemia & high leptin (N=149)** | | |  | **Low leptin/IGF-1/HbA1c (N=214)** | | |  |
| --- | --- | --- | --- | --- | --- | --- | --- | --- | --- | --- | --- | --- | --- | --- | --- | --- | --- | --- | --- | --- |
|  | **N** | **Median** | **Mean** | **SD** | **N** | **Median** | **Mean** | **SD** | **N** | **Median** | **Mean** | **SD** | **N** | **Median** | **Mean** | **SD** | **N** | **Median** | **Mean** | **SD** |
| **HbA 1c [%]** | 1281 | 4·8 | 4·7 | 0·6 | 449 | 4·7 | 4·7 | 0·5 | 474 | 4·8 | 4·7 | 0·6 | 149 | 4·8 | 4·7 | 0·5 | 209 | 4·6 | 4·5 | 0·6 |
| **TAG [mg/dl]** | 1225 | 45·0 | 56·2 | 24·2 | 443 | 45·0 | 54·9 | 24·9 | 452 | 45·0 | 50·8 | 11·6 | 144 | 83·0 | 87·0 | 36·6 | 186 | 45·0 | 48·8 | 12·6 |
| **HDL-c [mg/dl]** | 1225 | 53·0 | 53·1 | 14·3 | 443 | 55·0 | 57·6 | 12·8 | 452 | 53·0 | 53·4 | 13·2 | 144 | 35·5 | 37·2 | 10·3 | 186 | 53·5 | 53·7 | 14·3 |
| **CRP [ng/ml]** | 930 | 1289 | 7622 | 19353 | 326 | 643 | 1639 | 2484 | 320 | 3112 | 13811 | 26747 | 119 | 1734 | 9019 | 20291 | 165 | 1007 | 6431 | 16519 |
| **IL-6 [pg/ml]** | 886 | 0·3 | 0·6 | 2·8 | 305 | 0·2 | 0·2 | 0·2 | 312 | 0·4 | 0·8 | 1·2 | 111 | 0·4 | 0·5 | 0·5 | 158 | 0·3 | 0·9 | 6·4 |
| **Leptin [pg/ml]** | 892 | 1564 | 2838 | 4382 | 317 | 1518 | 2058 | 1828 | 305 | 1856 | 3430 | 5021 | 113 | 2382 | 5361 | 7612 | 157 | 880 | 1448 | 1927 |
| **IGF-1 [ng/ml]** | 1117 | 127·3 | 131·1 | 50·3 | 395 | 140·1 | 144·5 | 45·0 | 406 | 128·3 | 133·3 | 47·8 | 133 | 139·5 | 142·7 | 57·6 | 183 | 85·9 | 89·3 | 37·4 |
| **Ferritin [µg/l]** | 935 | 28·0 | 33·0 | 21·5 | 336 | 22·0 | 23·8 | 11·0 | 343 | 33·0 | 38·7 | 21·2 | 105 | 30·0 | 40·0 | 33·8 | 151 | 30·0 | 35·7 | 22·0 |
| **FU status** | **N** | **Median** | **Mean** | **SD** | **N** | **Median** | **Mean** | **SD** | **N** | **Median** | **Mean** | **SD** | **N** | **Median** | **Mean** | **SD** | **N** | **Median** | **Mean** | **SD** |
| **HbA 1c [%]** | 1247 | 5·0 | 5·0 | 0·3 | 543 | 5·0 | 5·0 | 0·3 | 261 | 5·0 | 5·0 | 0·3 | 209 | 5·0 | 5·0 | 0·3 | 234 | 4·9 | 4·9 | 0·3 |
| **TAG [mg/dl]** | 1279 | 57·0 | 65·4 | 31·1 | 557 | 55·0 | 59·9 | 22·1 | 271 | 54·0 | 57·4 | 17·6 | 211 | 99·0 | 102·3 | 44·5 | 240 | 51·0 | 55·0 | 22·4 |
| **HDL-c [mg/dl]** | 1279 | 60·0 | 60·4 | 13·7 | 557 | 62·0 | 64·3 | 12·0 | 271 | 60·0 | 61·4 | 11·9 | 211 | 43·0 | 44·0 | 7·0 | 240 | 64·0 | 64·6 | 13·1 |
| **CRP [ng/ml]** | 1173 | 280 | 1731 | 5217 | 497 | 173 | 408 | 555 | 249 | 953 | 4109 | 7609 | 202 | 590 | 3001 | 7714 | 225 | 169 | 877 | 3442 |
| **IL-6 [pg/ml]** | 1123 | 0·4 | 0·8 | 4·4 | 476 | 0·3 | 0·4 | 0·9 | 239 | 0·6 | 1·7 | 9·3 | 197 | 0·5 | 0·8 | 1·6 | 211 | 0·4 | 0·5 | 0·6 |
| **Leptin [pg/ml]** | 1180 | 4816 | 10382 | 14880 | 501 | 4345 | 6860 | 7321 | 250 | 8270 | 14761 | 18052 | 204 | 11296 | 20292 | 22561 | 225 | 2536 | 4376 | 6891 |
| **IGF-1 [ng/ml]** | 1214 | 289·6 | 295·7 | 116·1 | 530 | 327·0 | 317·3 | 109·5 | 259 | 290·6 | 293·9 | 106·4 | 200 | 345·6 | 335·7 | 124·0 | 225 | 188·9 | 211·2 | 91·0 |
| **Ferritin [µg/l]** | 1242 | 31·0 | 35·1 | 23·1 | 542 | 26·0 | 26·8 | 11·7 | 265 | 40·0 | 45·4 | 30·1 | 203 | 31·0 | 38·4 | 28·3 | 232 | 36·0 | 40·0 | 22·1 |

# **S8 Table:** Median, means and standard deviations for the concentrations of different biomarkers in the total analysis sample, in the four derived latent biomarker groups as well as by sex

(continued on next page)

| **Baseline**  **status** | **Boys (N=667)** | |  |  | **Girls (N=628)** | |  |  |
| --- | --- | --- | --- | --- | --- | --- | --- | --- |
|  | **N** | **Median** | **Mean** | **SD** | **N** | **Median** | **Mean** | **SD** |
| **HbA 1c [%]** | 656 | 4.8 | 4.7 | 0.6 | 625 | 4.7 | 4.6 | 0.6 |
| **TAG [mg/dl]** | 625 | 45.0 | 55.0 | 19.6 | 600 | 45.0 | 57.5 | 28.2 |
| **HDL-c [mg/dl]** | 625 | 52.0 | 53.2 | 14.3 | 600 | 53.0 | 52.9 | 14.2 |
| **CRP [ng/ml]** | 472 | 1155 | 7317 | 19528 | 458 | 1496 | 7936 | 19187 |
| **IL-6 [pg/ml]** | 449 | 0.3 | 0.5 | 1.0 | 437 | 0.3 | 0.6 | 3.9 |
| **Leptin [pg/ml]** | 452 | 1301 | 2184 | 3688 | 440 | 1929 | 3510 | 4911 |
| **IGF-1 [ng/ml]** | 574 | 114.9 | 118.4 | 46.2 | 543 | 141.8 | 144.6 | 51.0 |
| **Ferritin [µg/l]** | 481 | 27.0 | 31.7 | 23.3 | 454 | 30.0 | 34.4 | 19.3 |
| **Follow-up**  **Status** | **N** | **Median** | **Mean** | **SD** | **N** | **Median** | **Mean** | **SD** |
| **HbA 1c [%]** | 639 | 5.0 | 5.0 | 0.3 | 608 | 5.0 | 5.0 | 0.3 |
| **TAG [mg/dl]** | 659 | 54.0 | 62.5 | 31.4 | 620 | 61.0 | 68.6 | 30.6 |
| **HDL-c [mg/dl]** | 659 | 59.0 | 60.2 | 14.3 | 620 | 60.0 | 60.6 | 13.1 |
| **CRP [ng/ml]** | 614 | 268 | 1733 | 5172 | 559 | 287 | 1728 | 5269 |
| **IL-6 [pg/ml]** | 577 | 0.4 | 1.0 | 6.1 | 546 | 0.4 | 0.5 | 0.7 |
| **Leptin [pg/ml]** | 616 | 3054 | 7190 | 11617 | 564 | 7563 | 13869 | 17116 |
| **IGF-1 [ng/ml]** | 621 | 243.4 | 272.6 | 114.8 | 593 | 333.6 | 319.8 | 112.7 |
| **Ferritin [µg/l]** | 644 | 32.0 | 36.9 | 26.1 | 598 | 29.0 | 33.2 | 19.1 |

SD: standard deviation, T0: baseline, FU: follow-up, HbA1c: glycated hemoglobin A1c, TAG: triacylglycerides, HDL-c: high-densitiy lipoprotein cholesterol, CRP: C-reactive protein, IL-6: interleukin-6, IGF-1: insulin-like growth factor-1

Note: The LTA can handle missing data under a missing at random assumption. For this reason, biomarker data were not imputed.

# **S9 Table:** Transition probabilities (and 95% confidence intervals) from T0 to FU, i.e. probabilities to change from a certain biomarker status at T0 to another biomarker status at FU or to remain in the same biomarker status. Entries in bold font indicate membership in the same biomarker status at two consecutive time points.

| **Transition probabilities**  **from T0 to FU** | **Normal at FU** | **Inflammation at FU** | **Dyslipidemia & high leptin at FU** | **Low leptin/IGF-1/HbA1c at FU** |
| --- | --- | --- | --- | --- |
|  | Mean  (95% CI) | Mean  (95% CI) | Mean  (95% CI) | Mean  (95% CI) |
| **Normal at T0** | **72·8**  (58·4;86·8) | 5·5  (0·0; 18·5) | 14·0  (5·7; 23·0) | 7·7  (0·0; 19·1) |
| **Inflammation at T0** | 33·0  (13·5;50·7) | **58·4**  (38·5;77·6) | 6·0  (0·0; 16·2) | 2·6  (0·0; 21·2) |
| **Dyslipidemia & high leptin at T0** | 8·9  (0·0;25·5) | 5·5  (0·0; 24·6) | **73·7**  (54·4; 90·9) | 11·9  (0·0; 26·8) |
| **Low leptin/IGF-1/HbA1c at T0** | 10·0  (0·0;44·2) | 0·0  (0·0; 0·0) | 0·2  (0·0;3·9) | **89·8**  (55·7; 100) |

FU: follow-up

95% CI: 95% confidence interval; bias-corrected bootstrap confidence intervals estimated using 5000 replicates (sample size: N=1295)

|  | **Inflammation (ref: Normal)** | | | | **Dyslipidemia & high leptin (ref: Normal)** | | | | **Low leptin/IGF-1/HbA1c (ref: Normal)** | | | | |
| --- | --- | --- | --- | --- | --- | --- | --- | --- | --- | --- | --- | --- | --- |
| **Exposure** | **OR** | **LCL** | **UCL** | **p-value** | **OR** | **LCL** | **UCL** | **p-value** | **OR** | **LCL** | **UCL** | **p-value** |  |
| **Age** | 0·92 | 0·83 | 1·02 | 0·095 | 0·91 | 0·81 | 1·02 | 0·105 | **0·81** | **0·72** | **0·92** | **0·001** |  |
| **Female sex (ref: male)** | 0·76 | 0·52 | 1·10 | 0·146 | 0·76 | 0·50 | 1·16 | 0·202 | 0·99 | 0·63 | 1·56 | 0·956 |  |
| **Female sex*age** | 1·04 | 0·97 | 1·11 | 0·299 | 1·02 | 0·95 | 1·10 | 0·527 | 1·03 | 0·96 | 1·11 | 0·387 |  |
| **ISCED of parents (low/medium; ref: high)** | **1·57** | **1·12** | **2·20** | **0·009** | **1·72** | **1·17** | **2·51** | **0·005** | 0·79 | 0·53 | 1·19 | 0·257 |  |
| **ISCED of parents*age** | **1·07** | **1·00** | **1·15** | **0·041** | **1·12** | **1·04** | **1·20** | **0·002** | 1·04 | 0·96 | 1·11 | 0·339 |  |
| **Familial hypertension (yes; ref: no)** | 1·03 | 0·62 | 1·70 | 0·910 | 1·34 | 0·76 | 2·37 | 0·312 | **0·42** | **0·23** | **0·77** | **0·005** |  |
| **Familial hypertension*age** | 1·03 | 0·93 | 1·13 | 0·591 | 1·01 | 0·92 | 1·11 | 0·818 | 1·00 | 0·91 | 1·10 | 0·973 |  |
| **Familial diabetes (yes; ref: no)** | 0·78 | 0·29 | 2·11 | 0·630 | 0·81 | 0·26 | 2·51 | 0·714 | **0·23** | **0·07** | **0·78** | **0·018** |  |
| **Familial diabetes*age** | 1·06 | 0·88 | 1·29 | 0·533 | 1·07 | 0·88 | 1·30 | 0·524 | 1·03 | 0·84 | 1·25 | 0·786 |  |
| **Familial dyslipidemia (yes; ref: no)** | 1·09 | 0·58 | 2·06 | 0·782 | 1·38 | 0·67 | 2·84 | 0·380 | 0·66 | 0·30 | 1·43 | 0·294 |  |
| **Familial dyslipidemia*age** | 1·02 | 0·90 | 1·15 | 0·788 | 1·09 | 0·96 | 1·24 | 0·162 | 1·05 | 0·93 | 1·19 | 0·445 |  |
| **Maternal BMI** | 1·03 | 0·99 | 1·08 | 0·122 | **1·05** | **1·00** | **1·10** | **0·050** | **0·92** | **0·87** | **0·97** | **0·002** |  |
| **Maternal BMI*age** | 1·00 | 1·00 | 1·01 | 0·409 | **1·01** | **1·00** | **1·02** | **0·040** | 1·00 | 0·99 | 1·01 | 0·984 |  |
| **Birth weight (1 unit~100g)** | 0·98 | 0·94 | 1·01 | 0·135 | 0·99 | 0·95 | 1·03 | 0·627 | 1·01 | 0·97 | 1·06 | 0·506 |  |
| **Birth weight*age** | 1·00 | 0·99 | 1·01 | 0·800 | 1·00 | 1·00 | 1·01 | 0·368 | 1·00 | 0·99 | 1·01 | 0·740 |  |
| **Breast-feeding duration (1 unit~1 month)** | 1·01 | 0·98 | 1·04 | 0·688 | **1·05** | **1·01** | **1·08** | **0·011** | 1·04 | 1·00 | 1·08 | 0·056 |  |
| **Breast-feeding duration*age** | 1·00 | 0·99 | 1·00 | 0·776 | 1·00 | 0·99 | 1·00 | 0·312 | 1·00 | 0·99 | 1·00 | 0·334 |  |
| **Fruit/vegetable consumption** | 1·08 | 0·98 | 1·18 | 0·119 | 1·08 | 0·98 | 1·19 | 0·104 | 1·07 | 0·97 | 1·18 | 0·164 |  |
| **Fruit/vegetable consumption*age** | 1·00 | 0·98 | 1·02 | 0·822 | **0·98** | **0·96** | **0·99** | **0·010** | 1·00 | 0·98 | 1·02 | 0·731 |  |
| **Processed food consumption** | 1·04 | 0·85 | 1·26 | 0·715 | 1·13 | 0·92 | 1·39 | 0·230 | 1·13 | 0·91 | 1·41 | 0·275 |  |
| **Processed food consumption*age** | 1·04 | 1·00 | 1·09 | 0·066 | 1·00 | 0·96 | 1·05 | 0·926 | 1·00 | 0·95 | 1·04 | 0·854 |  |
| **Number of media (≥1; ref: 0)** | 1·30 | 0·91 | 1·85 | 0·143 | 1·16 | 0·77 | 1·72 | 0·479 | 0·82 | 0·55 | 1·23 | 0·329 |  |
| **Number of media*age** | 1·04 | 0·96 | 1·13 | 0·327 | **1·11** | **1·02** | **1·22** | **0·021** | 0·97 | 0·88 | 1·06 | 0·508 |  |
| **Well-being score (1 unit~10 points)** | 0·74 | 0·53 | 1·02 | 0·066 | 0·78 | 0·55 | 1·11 | 0·164 | 0·97 | 0·67 | 1·41 | 0·892 |  |
| **Well-being score*age** | 1·00 | 0·93 | 1·09 | 0·907 | **0·92** | **0·84** | **1·00** | **0·041** | 0·96 | 0·88 | 1·05 | 0·384 |  |
| **Sports club (yes; ref: no)** | **0·70** | **0·52** | **0·94** | **0·018** | **0·58** | **0·43** | **0·80** | **0·001** | 1·04 | 0·74 | 1·45 | 0·831 |  |
| **Sports club*age** | 0·96 | 0·89 | 1·04 | 0·313 | 0·95 | 0·88 | 1·03 | 0·230 | 0·95 | 0·88 | 1·03 | 0·233 |  |
| **Entered puberty (yes; ref: no)** | 1·08 | 0·28 | 4·17 | 0·915 | 1·67 | 0·41 | 6·75 | 0·473 | 0·26 | 0·06 | 1·14 | 0·075 |  |
| **Entered puberty*age** | 0·93 | 0·73 | 1·19 | 0·584 | 0·94 | 0·73 | 1·21 | 0·626 | 1·08 | 0·83 | 1·41 | 0·564 |  |
| **BMI z-score of child** | 1·14 | 0·99 | 1·32 | 0·071 | **1·54** | **1·32** | **1·81** | **<.0001** | **0·32** | **0·27** | **0·38** | **<.0001** |  |
| **BMI z-score*age** | **1·08** | **1·04** | **1·12** | **<.0001** | **1·12** | **1·08** | **1·16** | **<.0001** | 1·03 | 0·99 | 1·07 | 0·098 |  |

S10 Table: Odds Ratios and 95% confidence intervals for the unadjusted associations of modifiable and non-modifiable factors with the risk of showing the three distinct biomarker statuses (reference status: normal (status 1)); back transformed results of the multivariate mixed effects models.

Continuous variables were centred (and rescaled) to: 8 years of age, 3500 g birth weight (1 unit~ 100g), 6 months of breast-feeding, maternal BMI of 23 kg/m^2^, eating processed food 0 times/day, eating fruits and vegetables 5 times/day, well-being score of 40 (1 unit~10 points).

Statistically significant results are shown in bold.

ISCED: International Standard Classification of Education; LCL: lower 95% confidence limit; OR: Odds Ratio; ref: reference category; UCL: upper 95% confidence limit

# **S11 Table:** Sensitivity analyses for breast feeding duration

Odds Ratios and 95% confidence intervals for the associations of (1) total breast-feeding duration (in months), (2) exclusive breast-feeding duration (in months) as well as (3) an indicator of ‘at least 6 months exclusive breast feeding’ (vs less than 6 month) on the risk of showing the three distinct biomarker statuses (reference status: normal (status 1)); back transformed results of the multivariate mixed effects models.

|  | **Inflammation (ref: Normal)** | | | | **Dyslipidemia & high leptin (ref: Normal)** | | | | **Low leptin/IGF-1/HbA1c**  **(ref: Normal)** | | | |
| --- | --- | --- | --- | --- | --- | --- | --- | --- | --- | --- | --- | --- |
| **Exposure** | **OR** | **LCL** | **UCL** | **p-value** | **OR** | **LCL** | **UCL** | **p-value** | **OR** | **LCL** | **UCL** | **p-value** |
| Total breast-feeding duration (1 unit~1 month) | 1.04 | 1.01 | 1.07 | 0.013 | 1.07 | 1.03 | 1.11 | <0.001 | 1.06 | 1.02 | 1.11 | 0.003 |
| Total breast-feeding duration*age | 1.00 | 0.99 | 1.01 | 0.871 | 1.00 | 0.99 | 1.00 | 0.390 | 1.00 | 0.99 | 1.00 | 0.392 |
| Exclusive breast feeding duration (1 unit~1 month) | 1.06 | 0.99 | 1.15 | 0.108 | 1.05 | 0.96 | 1.15 | 0.253 | 1.17 | 1.06 | 1.28 | 0.001 |
| Exclusive breast feeding duration*age | 0.99 | 0.98 | 1.00 | 0.168 | 0.99 | 0.98 | 1.01 | 0.304 | 1.00 | 0.98 | 1.01 | 0.623 |
| Exclusive breast feeding ≥6 months (ref: <6 month) | 1.29 | 0.82 | 2.04 | 0.271 | 1.12 | 0.66 | 1.88 | 0.675 | 1.63 | 0.93 | 2.86 | 0.089 |
| Exclusive breast feeding ≥6 months (ref: <6 month)*age | 0.95 | 0.87 | 1.03 | 0.215 | 0.94 | 0.86 | 1.02 | 0.151 | 0.98 | 0.90 | 1.07 | 0.659 |
|  |  |  |  |  |  |  |  |  |  |  |  |  |

LCL: lower 95% confidence limit; OR: Odds Ratio; ref: reference category; UCL: upper 95% confidence limit

Continuous variables were centred (and rescaled) to: 8 years of age, 6 months of total breast-feeding, 6 months of exclusive breast-feeding

Models with this three alternative breast feeding exposures were each adjusted for age, sex, birth weight, country of residence, ISCED level and maternal BMI.

# **References**

1. Bullinger M, Brutt AL, Erhart M, Ravens-Sieberer U, Group BS. Psychometric properties of the KINDL-R questionnaire: results of the BELLA study. *Eur Child Adolesc Psychiatry* 2008; 17 Suppl 1: 125-32.

2. Ravens-Sieberer U, Bullinger M. Kindl-R English questionnaire for measuring health-related quality of life in children and adolescents. Revised Version Manual. . Ulrike Ravens-Sieberer & Monika Bullinger; 2000.

3. Santaliestra-Pasias AM, Mouratidou T, Verbestel V, et al. Physical activity and sedentary behaviour in European children: the IDEFICS study. *Public Health Nutr* 2014; 17(10): 2295-306.

4. Cole TJ, Lobstein T. Extended international (IOTF) body mass index cut-offs for thinness, overweight and obesity. *Pediatr Obes* 2012; 7(4): 284-94.

5. United Nations Educational SaCOU. International Standard Classification of Education, ISCED 2011. Canada: UNESCO Institute for Statistics; 2012.

6. Carskadon MA, Acebo C. A self-administered rating scale for pubertal development. *J Adolesc Health* 1993; 14(3): 190-5.

7. Lanza ST, Collins LM, Lemmon DR, Schafer JL. PROC LCA: A SAS procedure for latent class analysis. *Struct Equ Modeling* 2007; 14(4): 671-94.

8. Lanza ST, Collins LM. A new SAS procedure for latent transition analysis: transitions in dating and sexual risk behavior. *Dev Psychol* 2008; 44(2): 446-56.
